# Photocatalytic CO_2_ Reduction over Cotton-like Blue C/TiO_2_ Nanotubes: Enhanced Performance via Structural Engineering

**DOI:** 10.3390/nano16010035

**Published:** 2025-12-25

**Authors:** Wenjing Wu, Zichao Yang, Min Zhang, Zhongjie Guan, Jianjun Yang

**Affiliations:** National & Local Joint Engineering Research Center for Applied Technology of Hybrid Nanomaterials, Henan University, Kaifeng 475001, China; wuwenjing@henu.edu.cn (W.W.); 104753231846@henu.edu.cn (Z.Y.); zm1012@henu.edu.cn (M.Z.); guanzj@henu.edu.cn (Z.G.)

**Keywords:** photocatalytic reduction of CO_2_, MOF derivatives, TiO_2_ nanotubes, oxygen vacancies

## Abstract

Photocatalytic reduction of carbon dioxide is a very effective strategy to address the energy crisis and greenhouse effect. TiO_2_ is a widely used semiconductor photocatalyst, which has excellent catalytic activity, excellent chemical stability and low toxicity. Nevertheless, TiO_2_ still has some inherent limitations, such as: wide band gap, high carrier recombination rate, and low adsorption activation ability for carbon dioxide. These drawbacks severely restrict its further application in the photocatalytic reduction of CO_2_. In this study, cotton-like blue C/TiO_2_ NTs are successfully synthesized through the in situ growth of TiO_2_ nanotubes on the MIL-125(Ti)-derived C/TiO_2_ precursor. The experimental results revealed that the CO production rate of the cotton-like blue C/TiO_2_ NTs was 1.84 times that of C/TiO_2_ and 3.78 times that of TiO_2_ nanotubes. These results clearly demonstrate that the cotton-like blue C/TiO_2_ NTs exhibit a broad spectral response, a large specific surface area, and an abundance of oxygen vacancies. This research provides new insights into the design of titanium dioxide-based photocatalytic materials and opens up a promising avenue for enhancing the performance of titanium dioxide in the photocatalytic reduction of carbon dioxide.

## 1. Introduction

The substantial consumption of fossil fuels has fulfilled the requirements of industrialization. However, this has also led to the rapid depletion of non-renewable resources and excessive increase in carbon dioxide emissions [1,2,3]. Reducing carbon dioxide emissions by improving the combustion efficiency of fossil fuels or exploring clean and renewable energy sources (such as wind energy, tidal energy and solar energy) has been proven to be an effective strategy [4,5]. But the conversion and utilization of carbon dioxide is a more effective strategy. The carbon dioxide resource conversion technology mainly involves biological and chemical technologies. Biological transformation encompasses the process of carbon dioxide fixation by green plants or algae through photosynthesis in nature, as well as non-photosynthetic methods that utilize microorganisms and high-energy electron sources to convert carbon dioxide into valuable biological products [6,7]. On the other hand, chemical conversion refers to the process of converting carbon dioxide into useful products through the breaking and forming of chemical bonds under specific reaction conditions. This technology overcomes the drawbacks of biological conversion, such as low efficiency, harsh conditions, and long cycles [7,8,9]. Chemical conversion technologies, such as catalytic hydrogenation of carbon dioxide [10], electrocatalytic reduction [11,12], and photocatalytic reduction [1,5], have been widely reported. These methods utilize the catalytic action of catalysts to capture and break the C=O bonds in carbon dioxide, thereby converting it into fuels or chemicals. Compared with catalytic hydrogenation technology, photocatalysis and electrocatalysis, as emerging catalytic techniques, demonstrate great potential due to their mild reaction conditions and simple system structures [13,14]. It is important to note that, unlike electrocatalysis, photocatalysis does not require additional electrical energy input, which reduces operating costs and possesses environmental and sustainability features. Therefore, it is expected to become a potential alternative to traditional fossil fuels in terms of energy conversion [15].

Up to now, numerous semiconductor photocatalysts have been utilized to convert carbon dioxide into high-value organic compounds through photo-reduction, including formic acid (HCOOH), methanol (CH_3_OH), formaldehyde (HCHO), methane (CH_4_), and ethane (C_2_H_6_). Some typical examples of these photocatalysts are titanium dioxide (TiO_2_) [16], ZnO [17], Cu_2_O [18], CdS [19], g-C_3_N_4_ [20], and metal–organic frameworks [21]. Among these photocatalysts, titanium dioxide is regarded as one of the most promising candidate materials. This is attributed to its environmental friendliness, non-toxicity, high catalytic activity, low cost, and excellent chemical stability [22]. However, commercial titanium dioxide has a relatively weak absorption capacity for visible light, low efficiency, a high recombination rate of photogenerated carriers, and a limited number of active sites. These factors severely restrict the application of photocatalytic reduction of carbon dioxide [23]. In order to solve these problems and prepare efficient titanium dioxide photocatalysts, various modification techniques such as morphology control [24], defect engineering [25], doping [26], heterojunction construction [27], loading cocatalysts [28], self-assembly, and template preparation [29] are used to improve its visible light utilization and photocatalytic reduction performance of CO_2_.

Titanium dioxide nanotubes (TiO_2_ NTs) with various microstructures of titanium dioxide have attracted much attention in areas such as environmental protection and energy conversion. This is mainly attributed to its large specific surface area and rapid charge transfer rate. At present, the research for synthesis of TiO_2_ nanotubes clearly shows a strong dependence on the commercial P_25_ raw material. On the contrary, studies using non-P25 precursors are relatively rare. Although TiO_2_ nanotubes synthesized from commercial product P25 possess excellent specific surface area and charge transport properties, they also have some limitations, such as insufficient visible light response and limited number of active sites. Therefore, exploring new non-P_25_ raw materials and developing a synthesis strategy for TiO_2_ nanotubes that can simultaneously achieve high visible light utilization efficiency and a large number of active sites has significant theoretical significance and practical value. This effort is of crucial significance in breaking through the existing technical barriers and expanding the practical applications of titanium dioxide in related academic and industrial fields.

Metal–organic frameworks (MOFs) are emerging porous materials that are synthesized through the coordination of metal centers with organic ligands. Their unique properties, such as adjustable pore size and shape, diverse structural configurations, and extensive functionalities, have attracted significant attention in the academic community. Among various metal–organic framework materials, titanium-based metal–organic framework materials stand out due to their high stability and relatively simple synthesis process. These characteristics make them frequently used as precursors for functional TiO_2_ materials [18]. The resulting nano-sized TiO_2_ has a large specific surface area and porous structure, which can increase the exposed area of active sites and facilitate the adsorption and transport of reactant molecules [19,20,21]. For example, Zheng et al. [22] reported Ti^3+^-TiO_2_ derived from Ti-MOF nanosheets, which exhibited extremely high stability under environmental conditions. Li et al. [30] successfully synthesized the mixed-phase TiO_2_ through a two-step calcination process using MIL-125(Ti), and applied it for the photocatalytic degradation of nitrobenzene. Shi et al. [31] synthesized the TiO_2_@C composite material through a two-step process and used it as an electrode material. Although there have been previous related studies, as far as we know, there is no public research report available on the use of titanium-based metal oxides derived from metal–organic framework materials to prepare titanium dioxide nanotubes (TiO_2_ NTs). This provides an opportunity for research in this field.

In this study, C/TiO_2_ was synthesized by calcining MIL-125(Ti) as the precursor at high temperature. Subsequently, the processes of alkali etching, dissolution and recrystallization enabled the in situ growth of titanium dioxide nanotubes (TNTs). This disk-shaped C/TiO_2_ substrate played a role in constructing the TNT structural platform, thereby forming C/TiO_2_ nanotubes similar to cotton. The experimental results show that the rate of carbon dioxide generation by the cotton-like blue C/TiO_2_ nanotubes is 1.84 times that of C/TiO_2_ and 3.78 times that of TiO_2_ nanotubes. These results clearly indicate that the cotton-like blue C/TiO_2_ nanotubes have a wide spectral response, a large specific surface area, and abundant oxygen vacancies. Furthermore, the photocatalytic mechanism of carbonaceous C/TiO_2_ nanoclusters for carbon dioxide reduction was elucidated. It was emphasized that the synergistic effect of oxygen vacancies and hierarchical nanostructures in enhancing catalytic activity.

## 2. Experimental

### 2.1. Chemical Materials

The chemicals used in the experiment were purchased from commercial suppliers without further treatment. They are: N,N-Dimethylformamide (DMF, ≥98.5%, Aladdin), Titanium(IV) butoxide (≥98%, CP), Terephthalic acid (TPA, ≥99%, Aladdin).

### 2.2. Synthesis of the MIL-125(Ti)

MIL-125(Ti) was prepared by the conventional solvothermal method [27]. 6 mmol terephthalic acid (H_2_BDC) was put into the reactor tank, followed by 18 mL N, N-dimethyl formamide (DMF) solution, 2.0 mL methanol (CH_3_OH) solution, and 0.52 mL tetrabutyl titanate (TBOT) solution, then stirred until completely dissolved. Subsequently, the solution was put into the sealed reactors and reacted at 150 °C for 24 h. The suspension was subjected to centrifugation after natural cooling, and then was rinsed three times each with dimethylformamide and methanol. The obtained white powder was dried at 60 °C for 12 h to remove the residual reagents, thereby obtaining MIL-125(Ti).

### 2.3. Synthesis of the C/TiO_2_

The MIL-125(Ti) powder was heated in an argon atmosphere at a heating rate of 5 °C per minute to 600 °C. It was maintained at this temperature for 4 h, and then cooled naturally to room temperature. The C/TiO_2_ sample obtained through the high-temperature annealing of MIL-125(Ti) was labeled as MT.

### 2.4. Synthesis of the C/TiO_2_ NTs

100 mg of MT was weighed and added into 60 mL of 0.5, 1, 1.5, 2, and 3 mol/L NaOH solutions, respectively. The mixtures were magnetically stirred for 6 h until uniformly dispersed, followed by a hydrothermal reaction at 200 °C for 12 h. These products are first rinsed with deionized water until they reach neutrality, then immersed in 100 mL of 0.1 mol/L hydrochloric acid solution for 12 h, then rinsed again to neutrality, and finally dried under vacuum at room temperature. The obtained solids were placed in a porcelain container, and heated at a rate of 10 °C per minute in a tube furnace to 400 °C, and then calcined in an argon atmosphere for 2 h to obtain cotton-like C/TiO_2_ nanotubes. These samples were labeled as MTT0.5/1/1.5/2/3 according to the concentration of sodium hydroxide, where MTT represents TiO_2_ nanotubes prepared by alkaline etching of MIL-125(Ti) derived TiO_2_.

### 2.5. Structural Characterization

The crystal structures of C/TiO_2_ were characterized by X-ray diffraction (XRD, D8-ADVANCE, Bruker, Saarbrucken, Germany, Cu Kα radiation, 2θ = 20~80°), with the working voltage and current set at 40 kilovolts and 30 milliamperes, respectively; the morphologies characteristics were analyzed using transmission electron microscopy and high-resolution transmission electron microscopy equipped with FFT (TEM, HRTEM, JEOL JEM-F200, Tokyo, Japan). The UV–Vis diffuse reflectance spectra (DRS) absorbance spectra were obtained with a UV–Vis diffuse reflectance spectrophotometer (Shimadzu, Kyoto, Japan, UV-2600), using BaSO_4_ as the reference sample. Specific surface area, pore size distribution, and the physical adsorption spectrum of carbon dioxide were measured by the BET surface area measurement method (Quadrascorb SI-4, Quantachrome, Boynton Beach, FL, USA). This measurement was accomplished through N_2_ adsorption–desorption isotherms. The surface chemical states of elements in different samples were analyzed through X-ray photoelectron spectroscopy and Auger electron spectroscopy (XPS and AES, Thermo ESCALAB 250 Xi, Waltham, MA, USA), reference to the standard binding energy of C 1 s at 284.6 eV. The oxygen vacancy was measured by electron paramagnetic resonance (EPR, Bruker A300-10/12, Billerica, MA, USA). The electrochemical measurement was performed on an electrochemical analyzer (CHI600E, Shanghai Chenhua, China) with three electrode cells at room temperature. The working electrode is composed of indium tin oxide glass and the corresponding prepared sample. The Na_2_SO_4_ aqueous solution (0.1 M) was used as an electrolyte and a 300 W Xe lamp (PLS-SXE300/300UV, Beijing PerfectLight, China) used as the light source. Photoluminescence spectra (PL) were recorded on a confocal laser Raman microscope (HORIBA FLuoroMax+, Kyoto, Japan) using a 310 nm excitation light source. The in situ FT-IR was tested on a Bruker Tensor II spectrometer (Billerica, MA, USA). The samples were placed into the in situ reaction chamber of the infrared spectrometer, and conduct a 1 h pre-treatment of the sample under a vacuum condition of 80 °C. The photocatalytic reaction process is as follows: CO_2_ and 0.5 mL of water are slowly injected. The adsorption–desorption equilibrium is reached after 1 h of adsorption, and then the reaction system is exposed to light. Infrared spectra were collected at different light exposure times, and the process of the catalyst catalyzing the reduction of CO_2_ was analyzed.

Thermogravimetric (TG) test: Synchronous thermal analyzer (TGA/DSC3+, METTLER TOLEDO, Swiss), TG curve is collected, temperature range is 25~800 °C, in N_2_ atmosphere.

### 2.6. Photocatalytic Reduction Reaction of CO_2_

The performance of photocatalytic CO_2_ reduction was tested in a custom-made quartz glass reactor with a volume of 0.3 L. A 20 mg sample of catalyst powder was placed on the surface of the small circular table at the bottom, and 1 mL deionized water was uniformly added around the circular table. After sealing with a quartz lid, CO_2_ gas was passed into the reactor for 1 h, and ensure that the interior of the reactor is completely filled with CO_2,_ then both ends of the reactor were sealed. A 300 W xenon lamp (PLS-SXE300/300UV, PerfectLight, Beijing) was used as the lamp source, the product analysis by gas chromatograph (GC-2018, Shimadzu, Japan). The xenon lamp illuminates the reactor from the top and through the quartz glass cover at the bottom. Every hour, 1 mL of gas is collected from the reactor and injected into the gas chromatograph for quantitative detection of carbon monoxide and methane. By testing the standard gas, we obtained the retention time and the standard curve for each component gas.

## 3. Results and Discussion

### 3.1. The Structure and Morphology

**Figure 1:**
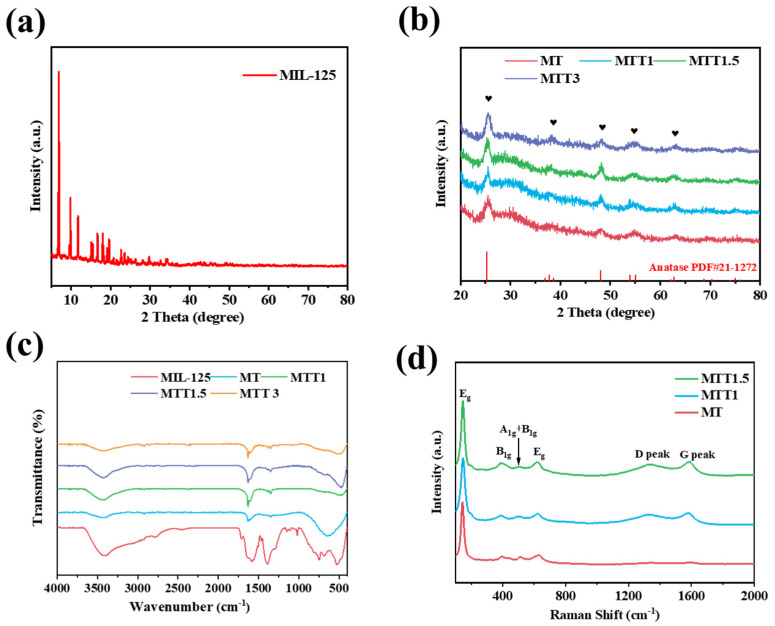
(**a**) XRD spectra of MIL-125(Ti). (**b**) XRD patterns and (**c**) FT-IR spectra of the synthesized catalysts. (**d**) Raman spectra of the synthesized catalysts.

The crystal phase structure of the catalyst was characterized using XRD (X-ray diffraction) technology. In Figure 1a, the diffraction peaks of the synthesized product were in complete agreement with the characteristic peaks of MIL-125(Ti) reported in the literature [32]. This result confirmed the successful synthesis of this metal–organic framework material. As presented in Figure 1b, after high-temperature calcination, the characteristic diffraction peaks of the original material completely disappeared. This indicated that the organic ligands underwent thermal decomposition, resulting in the formation of amorphous carbon. The newly emerged characteristic peaks corresponded to anatase-type TiO_2_ (JCPDS No. 21-1272). This finding revealed that during the thermal decomposition process, the MIL-125(Ti) precursor underwent a structural transformation to the anatase crystal form. After being treated with alkali, MTTx still retains the anatase crystal structure.

The Fourier transform infrared spectroscopy (FT-IR) spectrum of MIL-125(Ti) is presented in Figure 1c. The composite band spanning from 1470 to 1290 cm^−1^ and the band in the range of 1700–1500 cm^−1^ can be, respectively, ascribed to the symmetric and asymmetric stretching vibrations of carboxyl groups, as reported in reference [33]. In the region of 800–400 cm^−1^, the peaks are associated with the stretching vibration of Ti-O-Ti, This thus confirmed the existence of titanium oxide clusters. After undergoing high-temperature carbonization treatment, those obvious sharp peaks disappeared, while a broad shoulder area emerged at the same time. This proves the formation of TiO_2_.

As shown in Figure 1d, It is evident that all three samples exhibit vibration peaks at 146 cm^−1^, 395 cm^−1^, 515 cm^−1^, 630 cm^−1^. These peaks can be, respectively, attributed to the Eg, B1g, A1g + B1g, and Eg vibration modes of TiO_2_. This result is in accordance with the characteristic vibrational patterns of TiO_2_ in FT-IR. Meanwhile, in the high wavenumber region, the two peaks located at 1336 cm^−1^ and 1587 cm^−1^ are the characteristic peaks of the D band and G band of carbon, respectively. The D band is related to the vibration mode of sp^2^ hybridized carbon atoms in the hexagonal ring, indicating the presence of amorphous carbon. On the other hand, the G band originates from the stretching vibration of all sp^2^ atoms in the hexagonal ring and chain, representing graphitic carbon, and its appearance is due to the formation of nanocrystalline carbon [34].

**Figure 2:**
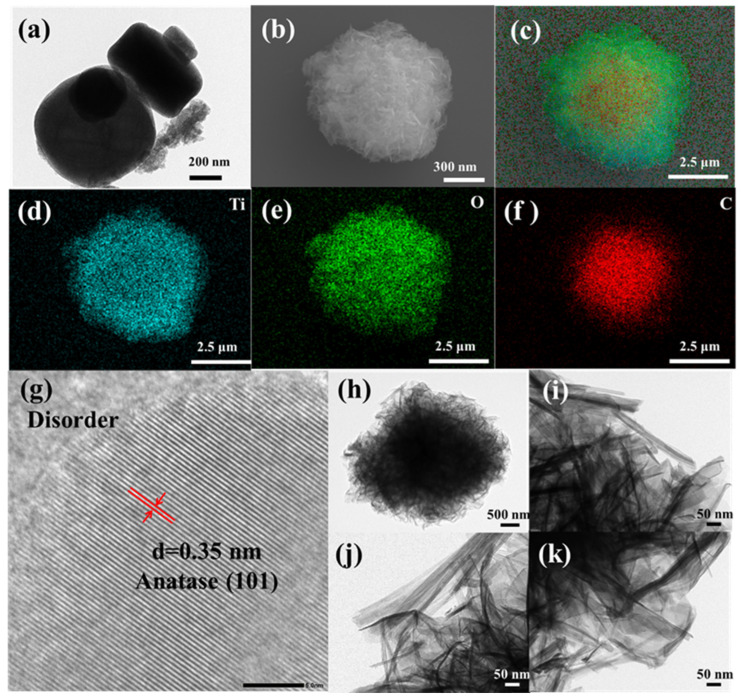
(**a**) TEM of the MT, (**b**–**f**) SEM and mapping of MTT1.5, (**g**) HRTEM and (**h**–**k**) TEM images of MTT1.5.

The surface morphology and structure of the catalyst were carefully analyzed, and the relevant results are shown in Figure 2. After thermal decomposition treatment, MIL-125(Ti) largely maintained its original shape. Obviously, the annealing process did not have a significant impact on the macroscopic structure of the photocatalyst. The disk-shaped structure of MT is composed of a large number of titanium dioxide particles, with each particle having a diameter of approximately 5 nanometers. Based on the X-ray diffraction analysis results, it can be determined that these particles are anatase-type titanium dioxide particles. As presented in Figure 2b, the morphology of MTT1.5 is that of a cotton-like disk-shaped structure, which is composed of nanotubes, nanowires, certain nanosheets, and accumulations of carbon frameworks. Compared with Figure 2a, it can be observed that the TiO_2_ particles in the MT after alkaline treatment have been etched into TiO_2_ nanotubes and nanosheets. The element distribution images in Figure 2c–f clearly show that Ti, O, and C are uniformly distributed within the catalyst, with C being encapsulated inside. This phenomenon indicates that the alkaline treatment led to the loss of carbon, and subsequently exposed the titanium dioxide. Notably, the HRTEM image clearly showed that the lattice fringes of the detected TiO_2_ had a size of 0.35 nm, which exactly corresponded to the interplanar spacing of the (101) plane of anatase TiO_2_.

**Figure 3:**
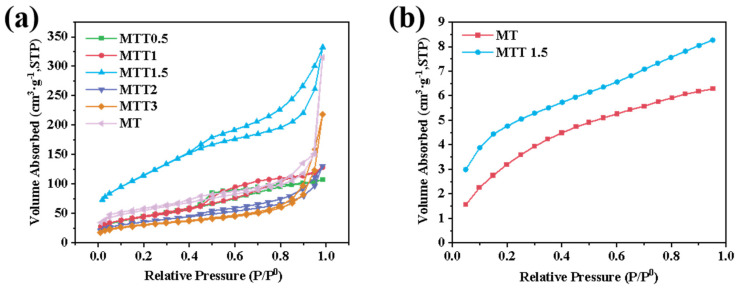
(**a**) N_2_ adsorption–desorption curve and (**b**) CO_2_ adsorption isotherms of synthesized catalysts.

The specific surface area of the catalyst was analyzed using a surface area and pore size analyzer. As shown in Figure 3a, according to the porosity Brunauer-Deming-Teller classification standard, all samples exhibited an IV-type isotherm, indicating the presence of typical mesoporous structures. From Figure 3b, compared with the MT samples, it can be seen that the alkaline treatment led to an increase in the number of mesopores or micropores. This was achieved through surface corrosion and the removal of some impurities that hindered the pores. For the samples treated with alkali, the average pore diameter decreased while the specific surface area increased. This change is more conducive to the adsorption of carbon dioxide. Among these samples, the specific surface area of MTT1.5 is the largest. However, further increasing the concentration of the alkaline treatment will lead to a decrease in the specific surface area. It is speculated that this might be due to the loss of mesoporous carbon in the MT samples during the alkaline treatment process. The average specific surface areas of MT, MTT1.5, and MTT3 are 76.27 m^2^/g, 172.0 m^2^/g, and 58.48 m^2^/g, while the average pore diameters are 10.18 nm, 4.919 nm, and 12.85 nm, respectively. The higher specific surface area of MTT1.5 makes it more conducive to the adsorption of carbon dioxide. As shown in the physical adsorption results of carbon dioxide in Figure 3b, this conclusion is further confirmed. The increase in specific surface area provides more pores and a larger surface area, which is beneficial for the physical adsorption of carbon dioxide. Therefore, MTT1.5 exhibits stronger carbon dioxide adsorption capacity, which is more conducive to the subsequent photoreduction reaction of carbon dioxide.

### 3.2. The Performances of Photocatalytic Reduction of Carbon Dioxide

**Figure 4:**
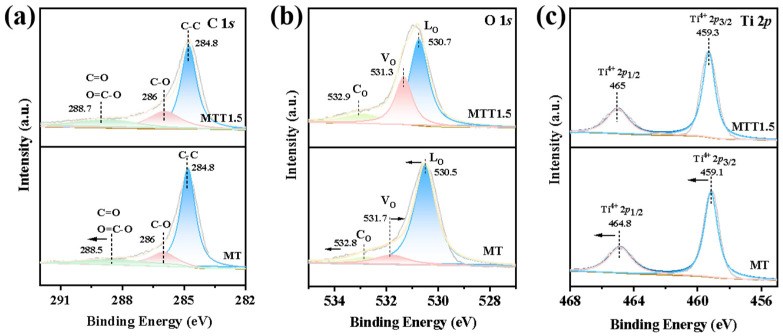
XPS spectra of the MT/MTT1.5 catalysts: (**a**) C 1s, (**b**) O 1s, (**c**) Ti 2p.

XPS (X-ray photoelectron spectroscopy) analysis was performed on TiO_2_ both before and after alkali treatment, and the outcomes are presented in Figure 4. In Figure 4a, the deconvoluted peaks of C 1s in the MT sample emerge at 284.8 eV, 286 eV, and 288.5 eV. These peaks correspond to sp^2^-hybridized C=C bonds, C-O bonds, as well as O-C=O and C=O bonds of the organic linkers [35]. In Figure 4b, the deconvoluted peaks of O 1s in the MT sample are located at 530.5 eV, 531.7 eV, and 532.8 eV, these peaks are associated with lattice oxygen, oxygen from surface hydroxyl group, and chemically adsorbed oxygen [36]. After the alkali treatment, the peak area at 530.5 eV decreased and it shifted towards a higher binding energy by 0.2 eV. The peak area at 531.7 eV increased and shifted to a lower binding energy by 0.4 eV. Additionally, the peak area at 532.8 eV showed a slight increase and shifted to a higher binding energy by 0.1 eV. This result indicates that lattice oxygen has indeed decreased. Specifically, the etching effect of sodium hydroxide causes the titanium-oxygen bonds on the surface to break, thereby generating oxygen vacancies and hydroxyl groups. In Figure 4c, the symmetrical peaks at 459.1 eV and 464.8 eV are ascribed to Ti 2p3/2 and Ti 2p1/2, respectively.

**Figure 5:**
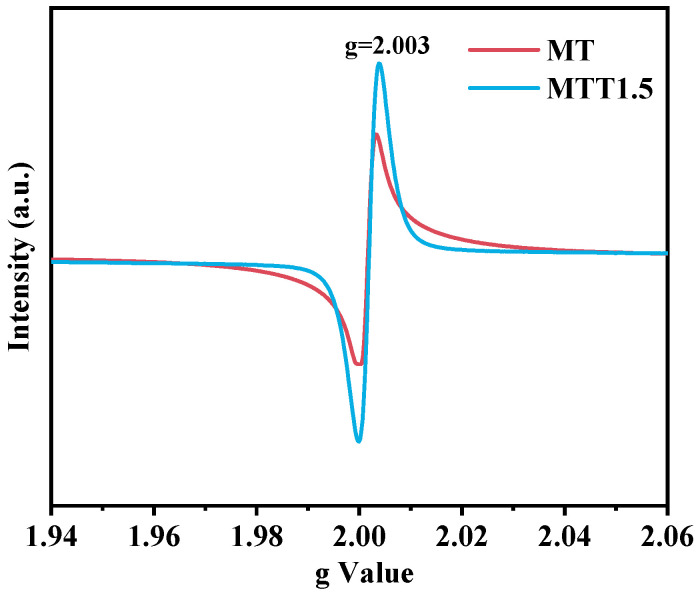
ESR spectra of samples MT and MTT1.5.

The electron paramagnetic resonance (EPR) measurements of the catalysts were conducted, and the relevant situation is shown in Figure 5. Both the MT and MTT1.5 samples exhibited strong EPR signals at g = 2.003. This characteristic peak can be attributed to oxygen vacancies, thereby providing strong evidence for the presence of oxygen vacancies in the samples.

**Figure 6:**
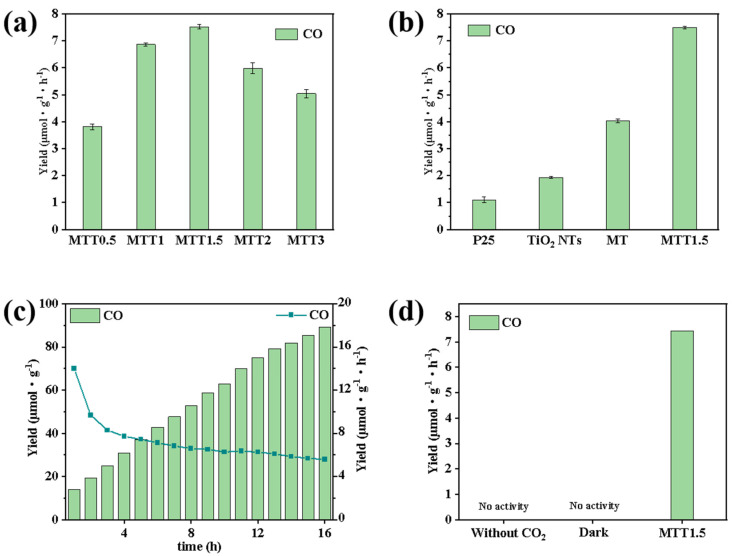
(**a**) CO_2_ photoreduction over Different NaOH concentrations. (**b**) Photocatalytic activity. (**c**) Cycling measurements for photoreduction CO_2_ of MTT1.5. (**d**) Photocatalytic activity of MTT1.5 for CO_2_ reduction under different atmospheres.

Under the same reaction conditions, the photocatalytic performance (for carbon dioxide reduction) of MTTx samples treated with different concentrations of sodium hydroxide was systematically evaluated. The results are shown in Figure 6a. The CO production from photocatalytic reduction of carbon dioxide shows a volcano-shaped trend. Specifically, when the sodium hydroxide concentration was 1.5 mol/L, the catalytic activity was the highest, with a CO production of 7.44 μmol·g^−1^·h^−1^. As the sodium hydroxide concentration increased further, the amount of carbon dioxide produced decreased. As shown in Figure 6b, the CO production of P_25_ and TiO_2_ nanotubes was 0.98 μmol·g^−1^·h^−1^ and 1.96 μmol·g^−1^·h^−1^, respectively. The CO production of MTT1.5 was 7.59 times that of P_25_, 3.79 times that of TiO_2_ nanotubes, and 1.84 times that of MTT. The stability of the MTT1.5 photocatalytic material was studied, as shown in Figure 6c. After continuous irradiation for 16 h, the CO production of this catalyst remained at 5.58 μmol·g^−1^·h^−1^, thus proving its excellent stability. To verify that the detected carbon dioxide was completely produced by the photocatalytic reduction of carbon dioxide, a control experiment was conducted under conditions without carbon dioxide and light. No reduction products were detected, which effectively confirmed the catalytic activity of MTT1.5 under light conditions.

**Figure 7:**
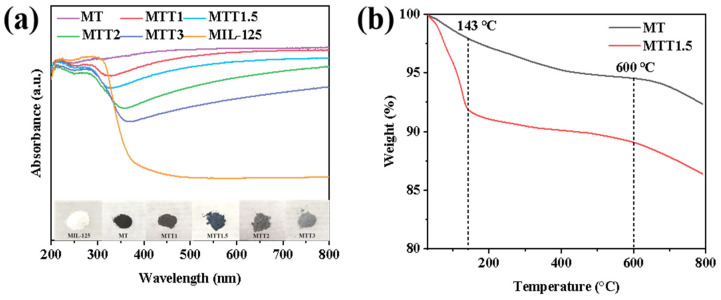
(**a**) UV–vis DRS and (**b**) TG curves of the synthesized catalysts.

The optical absorption properties of the catalyst were investigated by using the ultraviolet-visible diffuse reflectance spectroscopy (UV-vis DRS). As shown in Figure 7a, the MIL-125(Ti) material has relatively weak absorption ability in the visible light region (400–800 nm). Its optical absorption edge is located at approximately 380 nm, which is consistent with the typical semiconductor properties of Titanium-based metal–organic framework (MOF) materials. After undergoing high-temperature annealing treatment, the MT sample exhibited a significant improvement in its visible light absorption performance. This significant enhancement in light absorption performance can be attributed to the synergistic effect of the porous carbon framework formed during the high-temperature carbonization process and the Ti-O-C heterostructure. However, as the concentration of the base increases, the absorption intensity gradually decreases. The reduction in the number of carbon-based conjugated structures leads to a decline in their light absorption capacity. The thermal stability and composition of the materials were studied through thermogravimetric analysis. These materials were tested in a nitrogen environment, and the test results are shown in Figure 7b. As the temperature increased, MT showed a slight weight loss below 200 °C (3.27%), which might be attributed to the evaporation of adsorbed water or solvents. The MTT1.5 sample obtained after alkali treatment exhibited a similar weight loss pattern to MT within the medium-temperature range (150–500 °C) and the high-temperature range (500–800 °C). However, within the low-temperature range (50–150 °C), MTT1.5 showed a significant weight loss.

This might be due to the porous structure of this material. Such a structure makes it easy for it to absorb water or solvents from the environment, which leads to significant weight loss within the low-temperature range.

Furthermore, as the concentration of the alkali treatment increases, it can be clearly observed that the colors of the MT/MTT0.5/1/1.5/2/3 materials show a gradual change, from pure black, carbon gray, deep blue, dark gray, to light gray. This phenomenon is closely related with the decrease in the content of the carbonization product. Alkaline etching effectively removes the amorphous carbon on the surface, thereby exposing the TiO_2_ active layer rich in oxygen vacancies (Ov). The retained porous carbon framework and the oxygen vacancies together make to MTT1.5 present a stable blue color.

**Figure 8:**
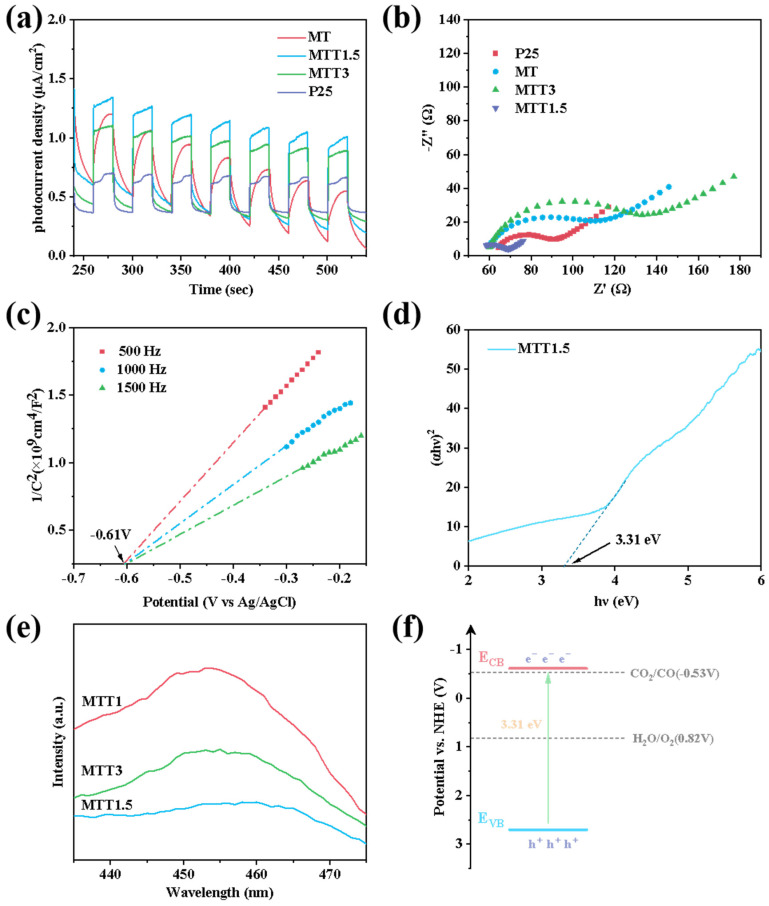
(**a**) Transient photocurrent curves and (**b**) Electrochemical impedance spectroscopy of synthesized catalysts. (**c**) Mott-Schottky diagram and (**d**) Bandgap of MTT1.5. (**e**) PL spectra of synthesized catalysts. (**f**) Band structure of MTT1.5.

The separation and migration efficiency of photogenerated carriers within the material were evaluated using photogalvanic response (I-t curve) and electrochemical impedance spectroscopy (EIS) techniques. The corresponding results are shown in Figure 8a,b. A periodic photogalvanic response was observed, where the current increased with light illumination and decreased when the light was turned off, indicating that the material has a stable ability to separate photogenerated carrier. The transient photogalvanic response and electrochemical impedance spectroscopy clearly showed that MTT1.5 had a higher photovoltaic response intensity and a smaller impedance spectrum arc radius. This means that MTT1.5 exhibited the best charge separation and migration ability among the samples studied. Combined with ultraviolet-visible diffuse reflectance spectroscopy tests, the band gap value was calculated in accordance with the semiconductor band gap formula:(αh*ν*)^n^ = k(h*ν* − E_g_). the band gap value of MTT1.5 is 3.31 electron volts, as shown in Figure 8d.

As shown in Figure 8c, the Mott-Schottky curves of MTT1.5 were measured at frequencies of 500 Hz, 1000 Hz, and 1500 Hz. The flat potential of the curve relative to the standard hydrogen electrode is approximately equivalent to the position of the conduction band bottom, which was determined to be −0.41 eV. Considering that the band gap of MTT1.5 is 3.31 eV, the position of the conduction band bottom was calculated as −0.61 eV (Vs. NHE), and the position of the valence band top is 2.70 eV, as shown in Figure 8f. This indicates that the conduction band electrons of MTT1.5 have the ability to reduce CO_2_ to CO.

As shown in Figure 8e, the carrier separation efficiency of the material was detected by steady-state fluorescence spectroscopy (PL) technology. The MTT1.5 catalyst showed the lowest PL signal intensity, indicating the lowest carrier recombination rate. The presence of an appropriate amount of oxygen vacancies can effectively capture photogenerated electrons, thereby improving the photocatalytic efficiency.

### 3.3. The Mechanism of Photocatalytic CO_2_ Reduction

**Figure 9:**
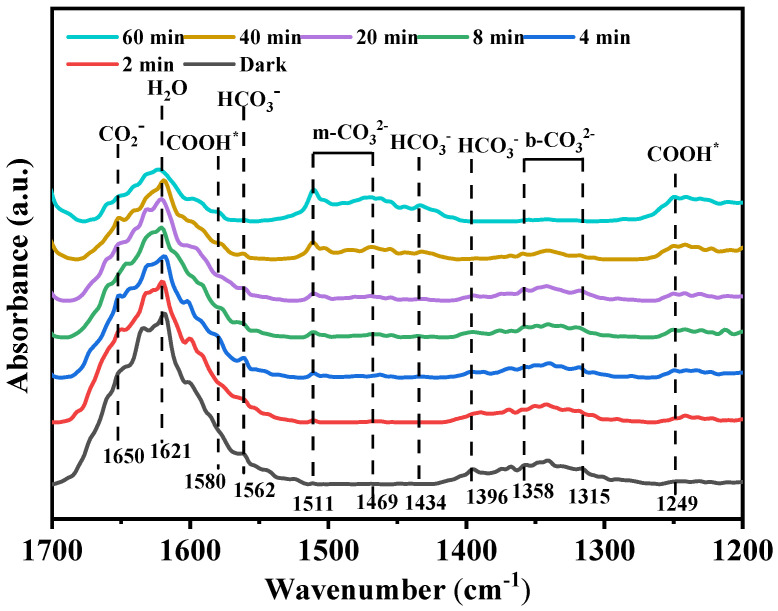
In situ DRIFTS spectra of the intermediate species of CO_2_ reduction on the MTT1.5.

As shown in Figure 9, a distinct absorption peak was observed within the range of 1650–1580 cm^−1^ after the dark-state adsorption. This peak can be attributed to the anti-symmetric stretching vibration of physically adsorbed CO_2_ or the O-H bending vibration of H_2_O. Under illumination, the decrease in peak intensity indicates that CO_2_ undergoes activation and participates in the photocatalytic reaction, while H_2_O, as a proton donor, is consumed. Furthermore, after the dark-state adsorption treatment, MTT1.5 exhibits an absorption band within the range of 1396 to 1315 cm^−1^. This absorption band is mainly attributed to the vibration modes of the bidentate carbonate. (b-CO_3_^2−^) and bicarbonate (HCO_3_^−^). This result is consistent with the surface reaction between CO_2_ and the hydroxyl groups on the catalyst, which leads to the formation of HCO_3_^−^ and CO_3_^2−^ species. The decrease in peak intensity under irradiation indicates that these intermediate products have been consumed during the subsequent reduction process. Under illumination, two new absorption peaks emerged in the range of 1511–1434 cm^−1^: The peak at 1511 cm^−1^ corresponds to the antisymmetric stretching vibration of monodentate species, which might be caused by the change in adsorption behavior under light exposure. This corresponds to the characteristic stretching vibration of the carbonyl group (C=O) in the carboxylic acid intermediate (COOH*). The peak at 1434 cm^−1^ is associated with the antisymmetric stretching vibration of HCO_3_^−^. The emergence of m-CO_3_^2−^ might be caused by the change in the adsorption configuration of CO_2_ on the catalyst surface. Furthermore, under light irradiation, a new peak at 1580 cm^−1^ emerged, which is the characteristic of the C=O stretching vibration of the carboxylic acid intermediate (COOH*). This judgment was further supported by the simultaneous presence of a vibrational peak at 1249 cm^−1^. Overall, these research results indicate that the main pathway for the generation of carbon monoxide is as follows: CO_2_ → HCO_3_^−^/CO_3_^2−^ → COOH* → CO.

**Figure 10:**
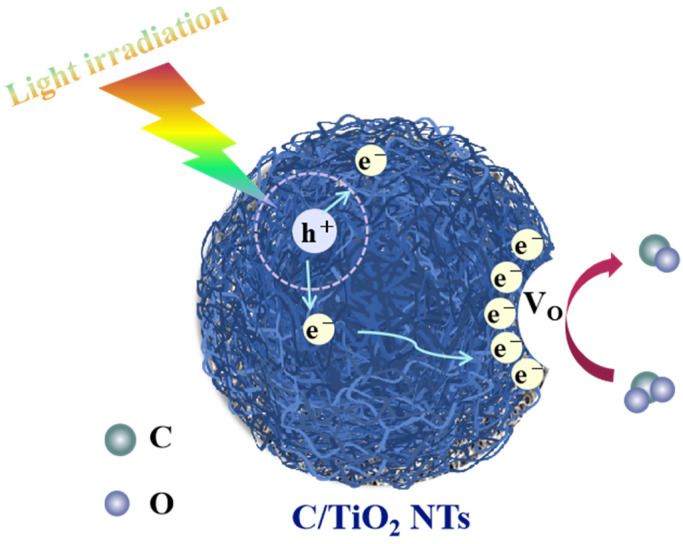
The photocatalytic reaction mechanism of CO_2_ on MTT1.5.

Based on the energy band positions of the cotton-like blue C/TiO_2_ nanotubes (NTs) and the above analysis, a catalytic mechanism for the photocatalytic reduction of carbon dioxide reaction was proposed, as shown in Figure 10. The existence of surface oxygen vacancies can effectively capture photogenerated electrons, thereby promoting the separation of charge carriers. Due to the more negative potential of the band center (CB) of TiO_2_, carbon dioxide can be reduced to carbon monoxide. Moreover, the carbon component plays a crucial role in improving the photocatalytic performance of carbon dioxide reduction. Specifically, the porous carbon structure provides abundant channels, promoting the physical adsorption of carbon dioxide. Additionally, the photogenerated electrons generated by the band center of TiO_2_ can easily transfer to the carbon, thereby inhibiting the recombination of charge carriers. Moreover, the carbon in the composite material can absorb long-wavelength visible light and near-infrared light, significantly enhancing the overall light capture ability of the material.

## 4. Conclusions

In this study, TiO_2_ nanotubes were successfully synthesized as a novel cotton-like blue carbon-modified TiO_2_ nanotube material by in situ growth on the carbon-doped TiO_2_ precursor of metal–organic framework MIL-125(Ti). Comprehensive experimental characterization confirmed the significant enhancement of the photocatalytic CO_2_ reduction performance of the synthesized material. Specifically, the CO yield of the cotton-like blue C/TiO_2_ nanotubes was 1.84 times higher than that of the C/TiO_2_ precursor and 3.78 times higher than that of traditional TiO_2_ nanotubes. These significant performance improvements are attributed to the unique structure and electronic properties conferred by the synthesis strategy. The cotton-like blue C/TiO_2_ nanotubes have: (1) an extended spectral response range to improve light capture; (2) a greatly increased specific surface area, providing abundant active sites; (3) high concentrations of surface defects, promoting charge separation and transfer efficiency. This strategy provides a promising approach for significantly improving the performance of TiO_2_ in sustainable energy conversion applications.

## Data Availability

The original contributions presented in this study are included in the article. Further inquiries can be directed to the corresponding author.
